# Characterization and validation of a ferroptosis-related LncRNA signature as a novel prognostic model for lung adenocarcinoma in tumor microenvironment

**DOI:** 10.3389/fimmu.2022.903758

**Published:** 2022-08-09

**Authors:** Yuanyong Wang, Guofang Lu, Xinying Xue, Mei Xie, Zhaoyang Wang, Zhiqiang Ma, Yingtong Feng, Changjian Shao, Hongtao Duan, Minghong Pan, Peng Ding, Xiaofei Li, Jing Han, Xiaolong Yan

**Affiliations:** ^1^ Department of Thoracic Surgery, Tangdu Hospital of Air Force Military Medical University, Xi’an, China; ^2^ Department of Physiology and Pathophysiology, National Key Discipline of Cell Biology, Fourth Military Medical University, Xi’an, China; ^3^ State Key Laboratory of Cancer Biology and National Clinical Research Center for Digestive Diseases, Xijing Hospital of Digestive Diseases, Fourth Military Medical University, Xi’an, China; ^4^ Department of Respiratory Disease, Beijing Shijitan Hospital, Capital Medical University, Peking University Ninth School of Clinical Medicine, Beijing, China; ^5^ Department of Respiratory Disease, School of Clinical Medicine, Weifang Medical University, Weifang, China; ^6^ Department of Respiratory and Critical Care, Chinese People's Liberation Army (PLA) General Hospital, Beijing, China; ^7^ Department of Oncology, Chinese People's Liberation Army General Hospital, Beijing, China; ^8^ Department of Thoracic Surgery, Xi’an International Medical Center Hospital, Xi’an, China; ^9^ Department of Ophthalmology, Tangdu Hospital of Air Force Military Medical University, Xi’an, China

**Keywords:** ferroptosis, lncRNA, lung adenocarcinoma, prognosis, tumor microenvironment

## Abstract

Ferroptosis is a more relatively recently identified type of programmed cell death, which is associated with tumor progression. However, the mechanism underlying the effect of ferroptosis-related long non-coding RNAs (lncRNAs) in lung adenocarcinoma (LUAD) remains elusive. Therefore, the current study aimed to investigate the role of ferroptosis-related lncRNAs in LUAD and to develop a prognostic model. The clinicopathological characteristics of patients and the gene sequencing data were obtained from The Cancer Genome Atlas, while the ferroptosis-associated mRNAs were downloaded from the FerrDb database. A ferroptosis-related lncRNA signature was established with Least Absolute Shrinkage and Selection Operator Cox regression analysis. Furthermore, the risk scores of ferroptosis-related lncRNAs were calculated and LUAD patients were then assigned to high- and low-risk groups based on the median risk score. The prognostic model was established by K-M plotters and nomograms. Gene set enrichment analysis (GSEA) was performed to evaluate the association between immune responses and ferroptosis-related lncRNAs. A total of 10 ferroptosis-related lncRNAs were identified as independent predictors of LUAD outcome, namely RP11-386M24.3, LINC00592, FENDRR, AC104699.1, AC091132.1, LANCL1-AS1, LINC-PINT, IFNG-AS1, LINC00968 and AC006129.2. The area under the curve verified that the established signatures could determine LUAD prognosis. The nomogram model was used to assess the predictive accuracy of the established signatures. Additionally, GSEA revealed that the 10 ferroptosis-related lncRNAs could be involved in immune responses in LUAD. Overall, the results of the current study may provide novel insights into the development of novel therapies or diagnostic strategies for LUAD.

## Introduction

Lung cancer is a type of cancer with high morbidity and mortality rates worldwide. It is estimated that approximately 25% of patients with lung cancer die from the disease, while the total overall 5-year survival rate is <20% (1). Non-small cell lung cancer (NSCLC) accounts for approximately 85% of all types of lung cancer, with adenocarcinoma, squamous carcinoma, adenosquamous carcinoma, large cell carcinoma and sarcomatoid carcinoma being the major pathological subtypes of NSCLC (2). Lung adenocarcinoma (LUAD) is the most common type of NSCLC (3). In addition, as a highly heterogeneous type of cancer with complex molecular mechanisms, lung cancer is resistant to several targeted therapies or the therapies are ineffective in certain patients, thus posing a substantial challenge in the treatment of lung cancer (4). Therefore, identifying specific carcinogenesis-related factors and novel biomarkers for the accurate diagnosis, individualized therapy and prognosis of LUAD is of significant importance.

Recently, ferroptosis has been identified as a unique type of iron-dependent programmed cell death, characterized by the accumulation of intracellular reactive oxygen species and lipoperoxide (5). It has been also reported that iron-dependent cell death serves a crucial regulatory role in tumor growth and is involved in the effectiveness of tumor radiotherapy and immunotherapy (6). Therefore, combining drugs targeting iron-dependent cell death-related signaling pathways can improve the anti-tumor efficacy of the above therapies. Chen et al. (7) demonstrated that the natural product erianin, isolated from Dendrobium gold, could induce iron-dependent cell death and exert anti-tumor effects in lung cancer cells *via* the Ca^2+^/CaM signaling pathway. Accumulating evidence has suggested that ferroptosis is associated with several biological processes in LUAD (8–10). However, the mechanisms underlying the regulation of ferroptosis remain elusive and are far from being exploited in cancer therapy. Therefore, identifying the crucial regulators of ferroptosis is a critical step for the therapeutic application of this process in cancer treatment.

Long non-coding RNAs (lncRNAs), non-coding (nc) RNAs >200 nucleotides in length, are not associated with protein translation, but they serve a vital role in gene regulation (11). More specifically, it has been suggested that the enhanced function or expression of lncRNAs can be involved in several diseases, including cancer. Emerging evidence has indicated that ferroptosis can be modulated by several lncRNAs. For example, Wang et al. (12) showed that LINC00618 could accelerate ferroptosis in an apoptosis-dependent manner in leukemia. Another study revealed that lncRNA PVT1 could regulate ferroptosis *via* the microRNA (miR)-214/transferrin receptor 1/p53 axis in acute ischemic stroke (13). Additionally, lncRNA MT1DP could enhance NSCLC sensitivity to promote ferroptosis *via* the miR-365a-3p/nuclear factor erythroid 2-related factor 2 axis (14). The above findings supported the critical role of lncRNAs in treating several types of cancer.

LncRNAs are involved in gene regulation *via* their direct binding with their target RNAs to regulate their translation or stability. It has been demonstrated that the above interactions are dependent on the binding of lncRNAs to their target RNAs, thus providing the appropriate substrates for protein functions or prohibitory protein effectors (15). Therefore, herein, the limma package, one of the analytical methods in the R language software, was used to perform a comprehensive analysis of gene expression profiles in LUAD (16). To identify ferroptosis-related lncRNAs associated with LUAD prognosis, co-expression and Cox and Least Absolute Shrinkage and Selection Operator (LASSO) regression analyses were performed. Furthermore, nomograms were established to evaluate the prognostic value of these lncRNAs and their capacity in predicting immune responses in LUAD. The results of the current study could assist in improving the diagnosis of LUAD at an early stage of the disease and in providing individualized treatment approaches for patients with LUAD.

## Materials and methods

### Datasets and patients

The clinical data and gene expression profiles from three LUAD datasets were downloaded from The Cancer Genome Atlas (TCGA) database. A total of 535 LUAD samples were included in the present study. Data exclusion criteria were as follows (1): h istologically confirmed LUAD and (2) available information on survival and gene expression. Finally, 513 patients with corresponding clinicopathological information were included for further study. A total of 513 LUAD patients were randomized 7:3 into a training cohort (359 patients) and a test cohort (154 patients).

### Identification of ferroptosis-related lncRNAs

Based on previous studies, ferroptosis-related mRNAs are listed in **Supplementary Table 1** (17–19). Firstly, the expression profile of lncRNAs and mRNAs were obtained from TCGA. Subsequently, the expression profile of ferroptosis-related lncRNAs were extracted from ferroptosis-related mRNAs using co-expression analysis (20). R was used to evaluate the association between the expression levels of lncRNAs and ferroptosis-related mRNAs in LUAD specimens. The association was determined using Pearson’s correlation coefficient analysis (P<0.05; r>0.40).

### Validation of risk score

The limma package, a commonly used R package, was utilized to analyze the differential expression of ferroptosis-related lncRNAs with a threshold of P<0.05 and absolute log2 (fold change) >1 (21). The overall survival (OS) rate was set as the clinical endpoint of the present study. Univariate Cox regression analysis was performed to establish the ferroptosis-related lncRNA model. Hazard ratio (HR) >1 was considered to indicate a significant association.

Furthermore, the association of the selected differentially expressed lncRNAs (DElncs) with prognosis were then investigated. The prognostic value of the selected DElncs was determined using LASSO Cox regression analysis provided by the glmnet R package (22). The risk scores of the key lncRNAs were obtained *via* measuring their expression levels and LASSO regression coefficients (23). Finally, the LUAD samples were used as training cohorts and divided for subsequent analyses into low- and high-risk groups, using the median risk score as the cut-off point.

The SurvivalROC and “survival” R packages were used to assess the power of the prognostic model (21). Additionally, the survival between the low- and high-risk groups was evaluated using the “survival” package and Kaplan-Meier log-rank test (24).

### Independent prognostic analysis and construction of nomogram

To evaluate whether the ferroptosis-related lncRNA model was independent from clinical factors, such as sex, age, smoking and clinical stage, and whether the model exhibited a significant value in predicting OS, univariate and multivariate Cox regression analysis was carried out in the LUAD datasets downloaded from TCGA. The independent prognostic factors were assessed by multivariate Cox regression analysis and a prognostic nomogram plot was constructed in R (25).

### Functional enrichment analysis

Gene ontology (GO) and Kyoto encyclopedia of genes and genomes (KEGG) pathway enrichment analyses were performed to determine the biological function of the selected lncRNAs (26). A heatmap was constructed to visualize the abundance of the selected DElncs in both groups of TCGA database.

### Immunogenomic landscape analyses

ESTIMATE (27) and TIMER (28), two bioinformatics analysis software, were used to estimate the immunogenomic functions, cell infiltration and expression of immune checkpoint molecules. The differences were compared with Wilcoxon test.

### Prediction of immunotherapy response and sensitivity to chemotherapy

The pRRophetic R package was utilized to predict chemosensitivity between the two risk groups in the LUAD cohorts. Several common anticancer drugs were identified, including AICAR, AKT inhibitor VIII, bicalutamide, bleomycin, cyclopamine, doxorubicin, epothilone B, etoposide, lapatinib, obatoclax, mesylate, parthenolide, PD.173074, pyrimethamine, salubrinal, shikonin and vinorelbine. The half-maximal inhibitory concentration (IC_50_) of the above drugs was determined (29, 30).

### Reverse transcription-quantitative PCR (RT-qPCR)

A total of 45 pairs of LUAD tissues were obtained from the Tangdu Hospital of Air Force Medical University. The present study was approved by the Ethics Committee of the Tangdu Hospital of Air Force Medical University. GAPDH served as the internal control for qPCR. The relative expression levels of 10 signature lncRNAs were calculated using the 2^-ΔΔCq^ method (31).

## Results

### Acquisition of ferroptosis-related lncRNAs and mRNAs

**Figure 1:**
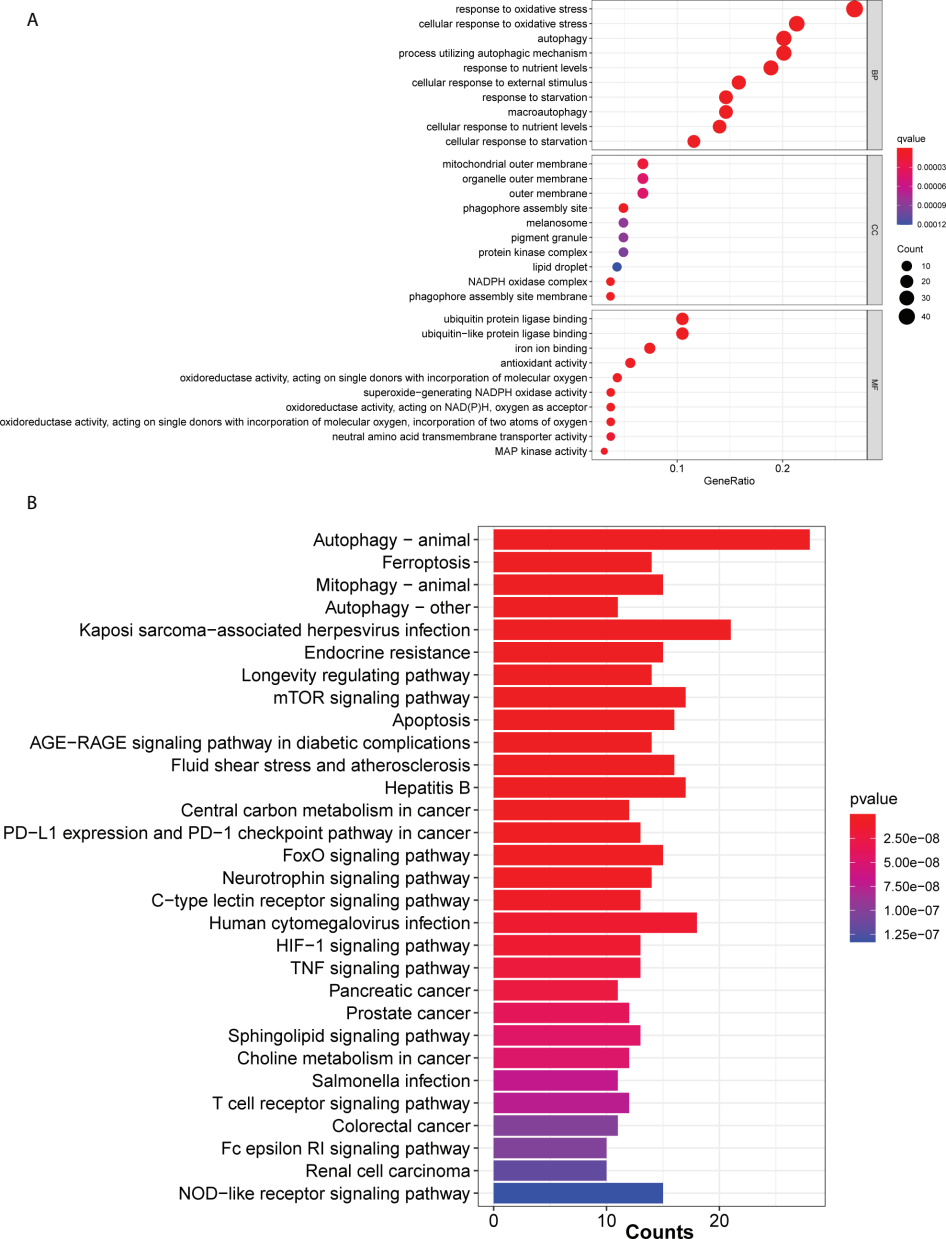
Ferroptosis-associated mRNAs in The Cancer Genome Atlas cohort. **(A)** Bar and **(B)** cluster plots of significantly enriched Gene Ontology and Kyoto Encyclopedia of Genes and Genomes pathways, respectively, are shown.

A total of 259 ferroptosis-related genes were obtained from FerrDb (**Supplementary Table 1**). GO and KEGG analysis of the ferroptosis-related mRNAs revealed that the majority of mRNAs were enriched in apoptosis-related processes such as autophagy, oxidative stress and ferroptosis (**Figure 1**). Additionally, the clinical characteristics and gene expression profiles in the LUAD cohort were downregulated in the datasets obtained from TCGA. To obtain significant lncRNAs, samples with low expression levels were excluded using a cut-off value of mean expression >0.5 across all samples. Subsequently, the association between the expression levels of ferroptosis-related mRNAs and lncRNAs in LUAD samples was assessed using the limma package in R language. The threshold for correlation was set as correlation coefficient >0.4 and P<0.05. Finally, a total of 2,420 ferroptosis-related lncRNAs were identified.

### Construction and validation of a prognostic ferroptosis-related lncRNA model in TCGA cohort

**Figure 2:**
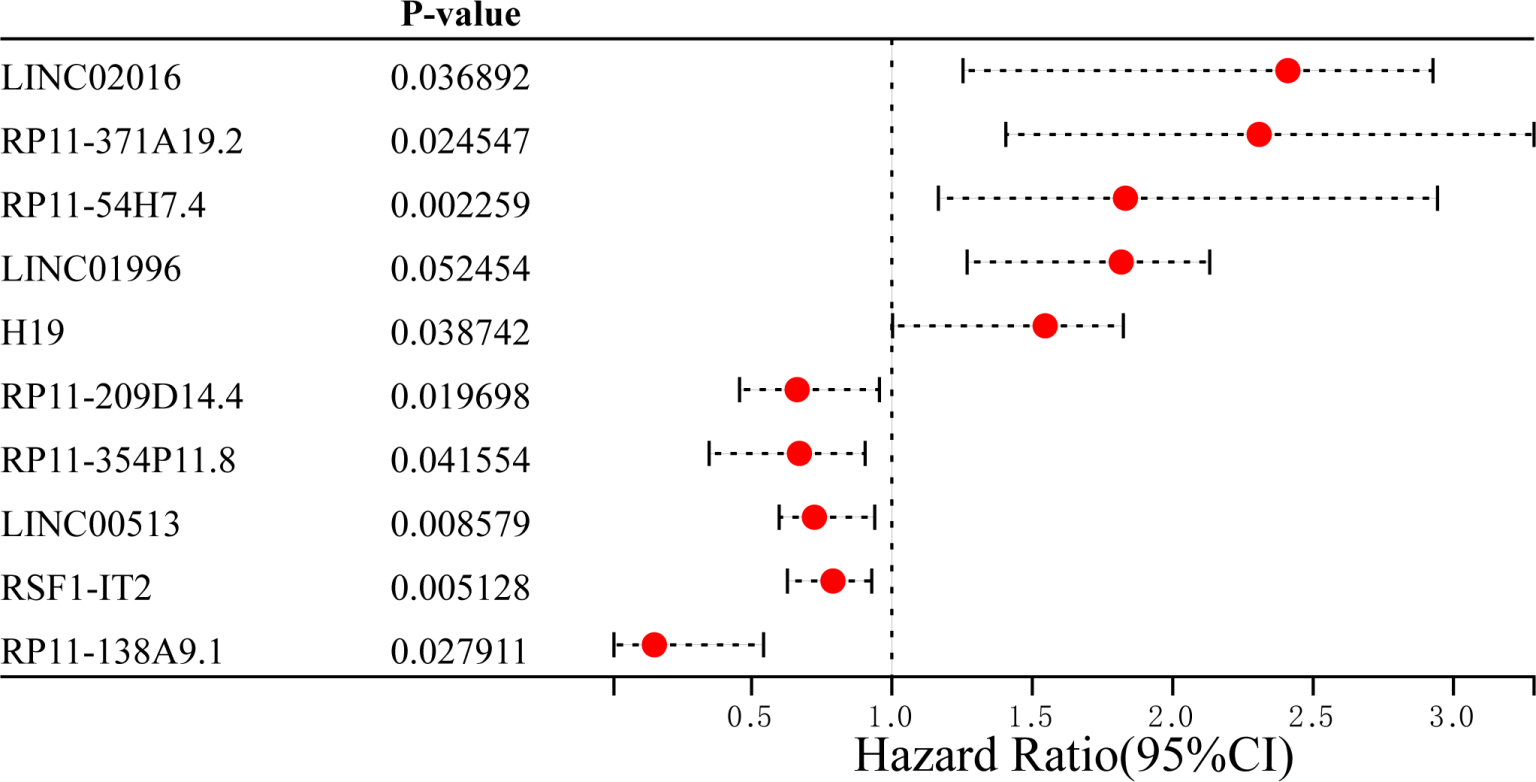
Top 5 differentially upregulated and downregulated ferroptosis-related long non-coding RNAs between lung adenocarcinoma and normal samples. HR, hazard ratio.

According to the LASSO Cox regression model, the top 10 significantly differentially expressed mRNAs are shown in **Figure 2** total of 10 ferroptosis-related lncRNAs associated with OS were obtained through distinctive formulas and the risk score for each individual was calculated. Based on the median risk score, the 359 LUAD patients were divided into the high- (n=179) and low-risk (n=180) groups.

**Figure 3:**
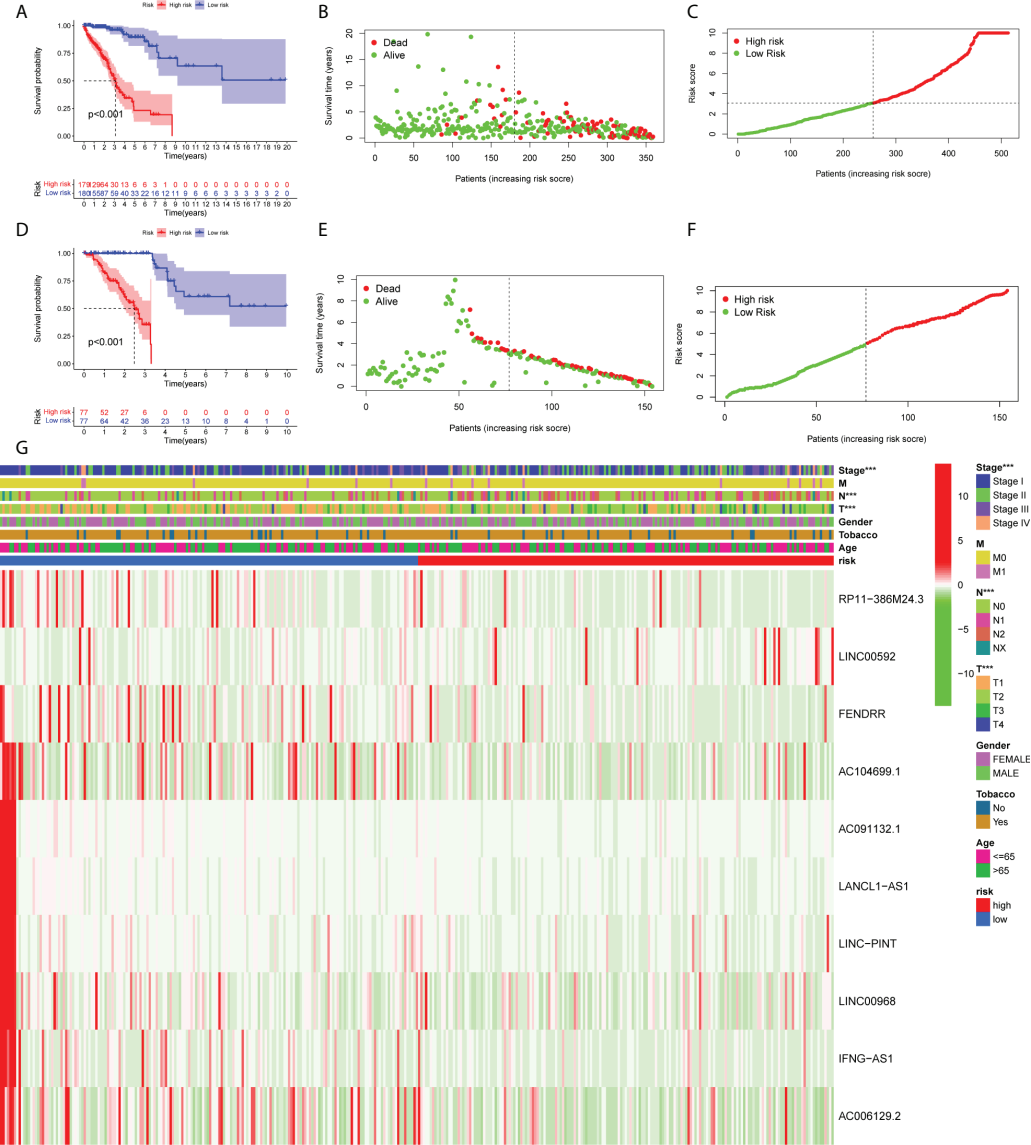
Development and validation of the prognostic ferroptosis-associated long non-coding RNA signature. **(A, D)** Kaplan-Meier curve, **(B, E)** risk score, **(C, F)** survival status and **(G)** heatmap are shown. ***means p<0.001.

As shown in **Figure 3A**, LUAD patients with low-risk scores exhibited a significantly poorer mortality rate. As the risk score was increased, the risk of mortality was also elevated, while the survival rate was reduced (**Figures 3B, C** **)**. The same results are shown in the validation cohort (**Figures 3D** **–** **F**). The risk heatmap illustrates the expression profile of lncRNAs between the high- and low-risk groups (**Figure 3G**). Additionally, the expression levels of 10 ferroptosis-related lncRNAs were notably associated with several clinical features, including clinical stage, T stage and N stage. The aforementioned results indicated that the expression levels of RP11-386M24.3, LINC00592, FENDRR, AC104699.1, AC091132.1, LANCL1-AS1, LINC-PINT, IFNG-AS1, LINC00968 and AC006129.2 were significantly different in the current prognostic model.

### Independent prognostic analysis of OS and construction of a predictive nomogram in LUAD

**Figure 4:**
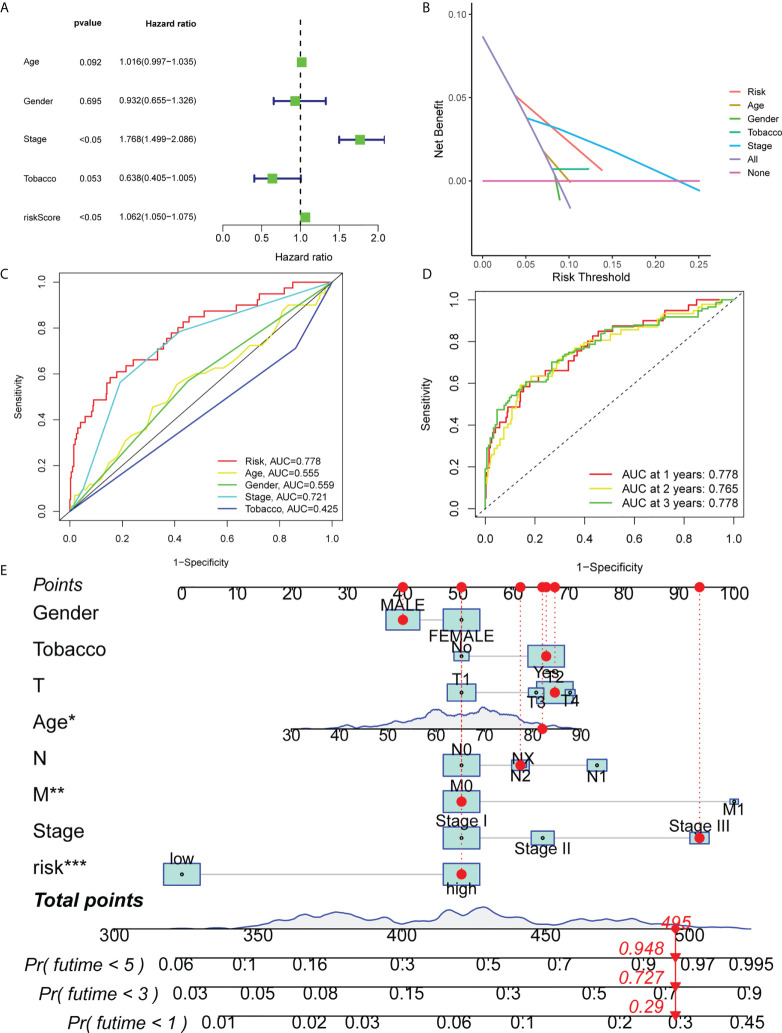
Independent prognostic factors for OS in LUAD. **(A)** Multivariate Cox regression analysis is shown. **(B)** Decision curve analysis is presented. **(C)** ROC curves of risk scores and other clinical characteristics based on OS are shown. **(D)** ROC curves predicting the OS of patients with LUAD at 1, 3 and 5 years are shown. **(E)** Nomogram validating the OS of patients with LUAD. OS, overall survival; ROC, receiver operating characteristic; LUAD, lung adenocarcinoma. *means p<0.05, **means p<0.01, ***means p<0.001.

Subsequently, the present study evaluated whether clinical features, such as age, sex, clinical stage, smoking history and risk scores were independent prognostic factors using multivariate Cox regression and decision curve analyses. The results demonstrated that clinical stage and risk score were independent predictive factors for OS (**Figures 4A, B** **)**. Furthermore, the diagnostic efficacy of other baseline factors and that of risk scores in LUAD patients were compared *via* calculating the area under the curve (AUC). AUC values of 0.778 and 0.721 were obtained in the model for risk scores and clinical stage, respectively (**Figure 4C**). The above values were higher compared with other parameters, thus verifying that the ferroptosis-associated lncRNA signature exhibited enhanced diagnostic value compared with other prognostic factors in LUAD patients. In **Figure 4D**, the survival rate for the first three years is presented. Additionally, a nomogram was constructed to predict OS in patients with LUAD based on independent predictors derived from a multivariate Cox risk regression analysis model (**Figure 4E**).

### Analysis of immunity based on N6-methyladenine (m^6^A) methylation and immune checkpoint inhibitors

**Figure 5:**
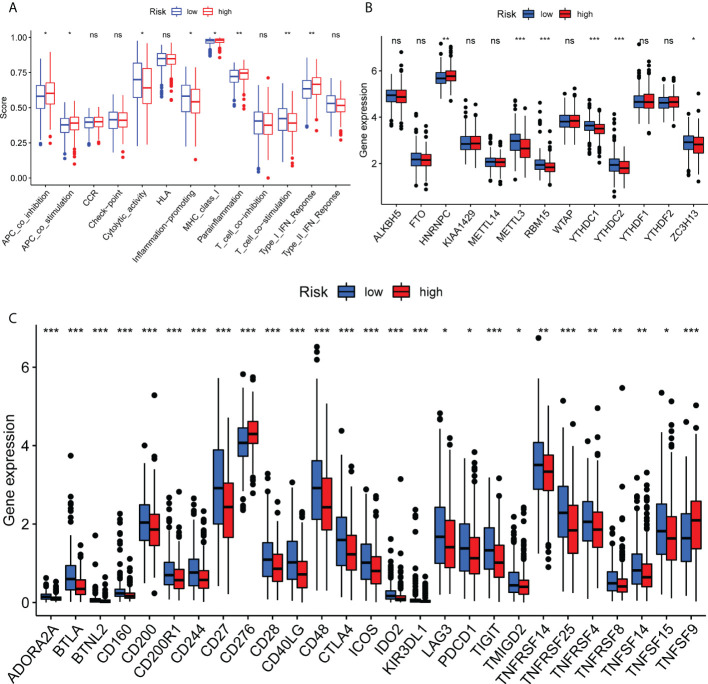
Immune function analysis of ferroptosis-related genes in The Cancer Genome Atlas in the lung adenocarcinoma cohort. **(A)** Single sample gene set enrichment analysis algorithms are shown. **(B)** Methylation levels are shown. **(C)** Check points are shown. *P<0.05, **P<0.01 and ***P<0.001. Ns means no significance.

Subsequently, the current study determined the infiltration rate of immune cells and immune-related functions in both risk groups using the XCELL, TIMER, MCP counter, CIBERSORTx, QUANTISEQ and EPIC tools, and single sample gene set enrichment analysis (ssGSEA) algorithms to evaluate the association between immune function and LUAD prognosis. As shown in **Figure 5**, a significant difference was obtained between the two risk groups. Additionally, significant differences were observed between the high- and low-risk groups in terms of immune function, including antigen-presenting cell co-stimulation, inflammation, cytolytic activity, parainflammation, T cell co-stimulation and type I IFN responses (P<0.05; **Figure 5A**). In addition, the expression levels of HNRNPC, METTL3, RBM15, YTHDC1, YTHDC2 and ZC3H13, key factors of m^6^A methylation, were notably decreased (**Figure 5B** **)**. Immunophenoscore analysis and the expression levels of immune modulators were used to predict the response of patients with LUAD to immune checkpoint inhibitors (ICIs). Therefore, the expression levels of the immune modulators CD160, CD200, CD244, CD27, CD276, CD28, PDCD1 and TIGIT were markedly reduced in the high-risk group compared with the low-risk group (P<0.05; **Figure 5C**). The aforementioned findings suggested that the ferroptosis-related prognostic signature could predict the possible efficacy of ICIs in patients with LUAD and could be considered as a classifier for treatment selection.

### Analysis of immune cell infiltration

**Figure 6:**
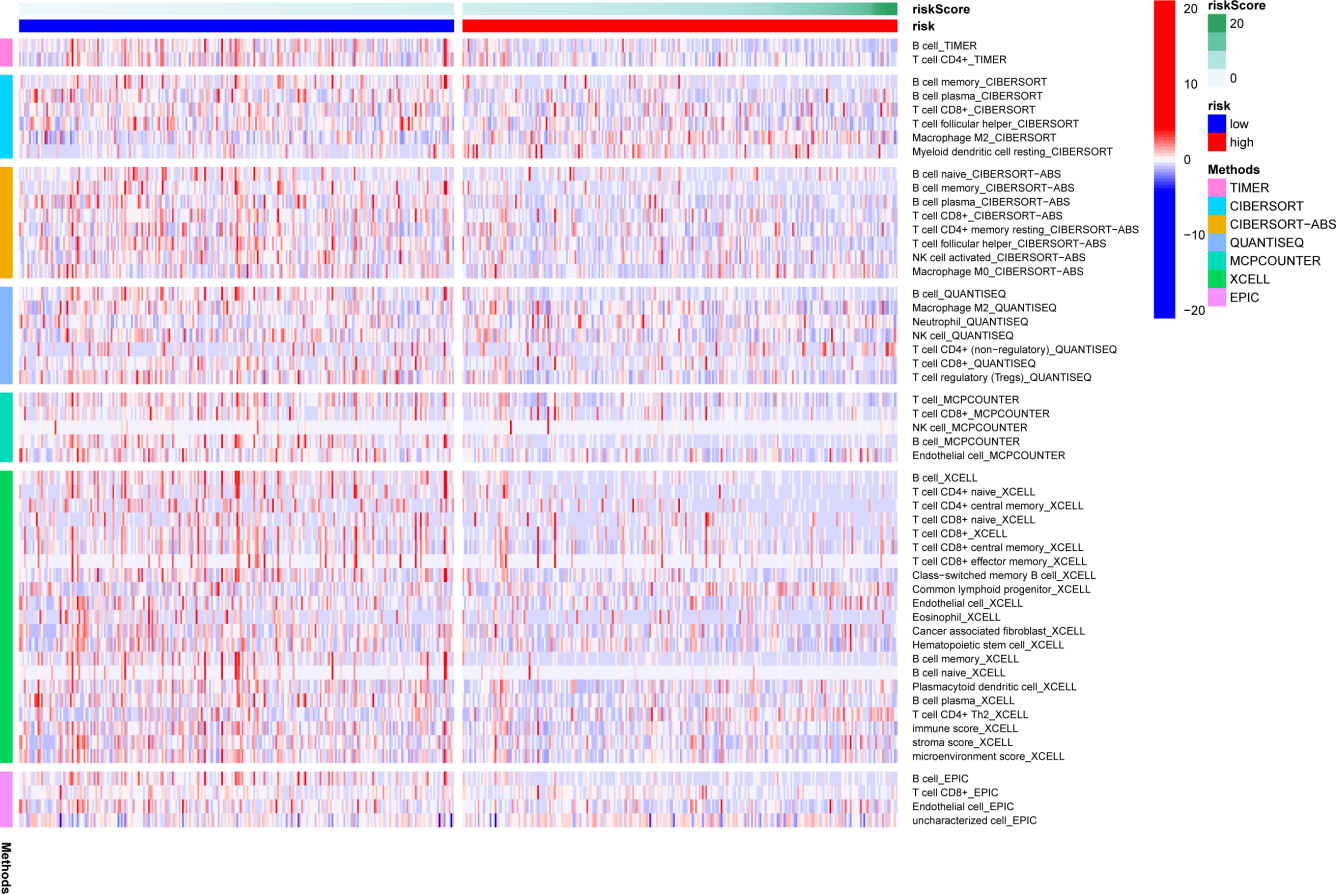
Comparison of immune analysis between both risk groups. The ESTIMATE, TIMER, MCP counter and CIBERSORTx algorithms were used to compare the cellular components or cell immune responses between the high- and low-risk groups. ns, not significant.

A heatmap of immune responses based on TIMER, CIBERSORT, CIBERSORT-ABS, QUANTISEQ, MCPcounter, XCELL and EPIC tools is shown in **Figure 6**. The results suggested that the risk score was negatively associated with immune cell infiltration in LUAD samples, with an immunoreactive status in the low-risk group.

### GSEA

**Figure 7:**
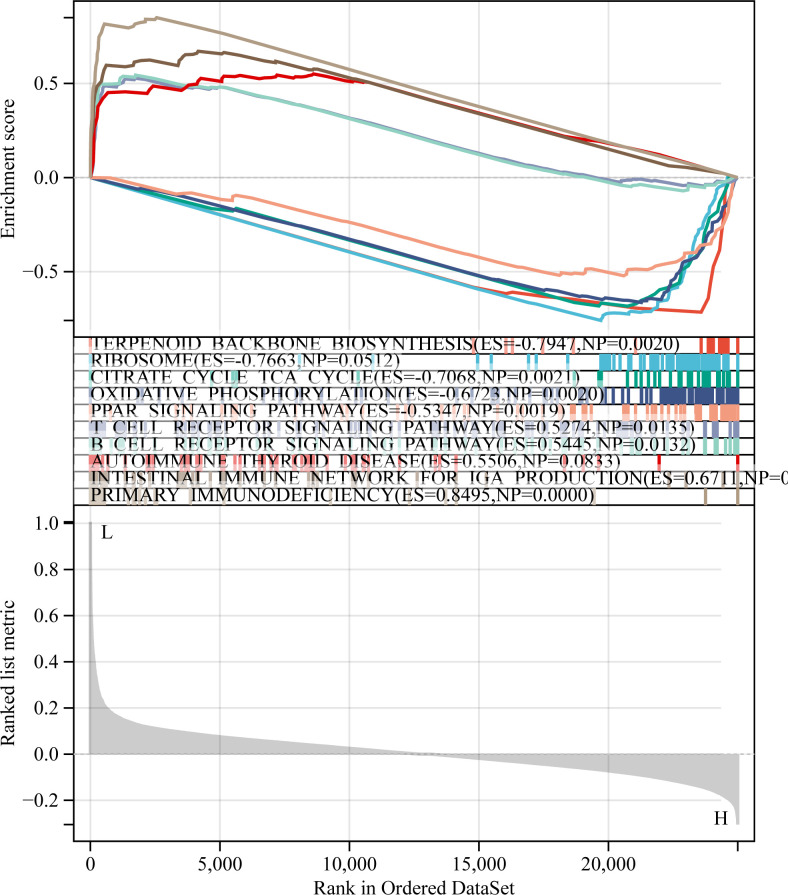
Gene set enrichment analysis is presented. P<0.05; False discovery rate <0.05.

A total of 10 ferroptosis-related lncRNAs were also associated with immune responses. This finding was further investigated *via* comparing the immune-related functions in GSEA between the two groups. The results demonstrated that immune system processes and immune response were markedly enriched in the low-risk group compared with the high-risk group (**Figure 7**).

### Ferroptosis-associated lncRNAs can predict the response to chemotherapy

**Figure 8:**
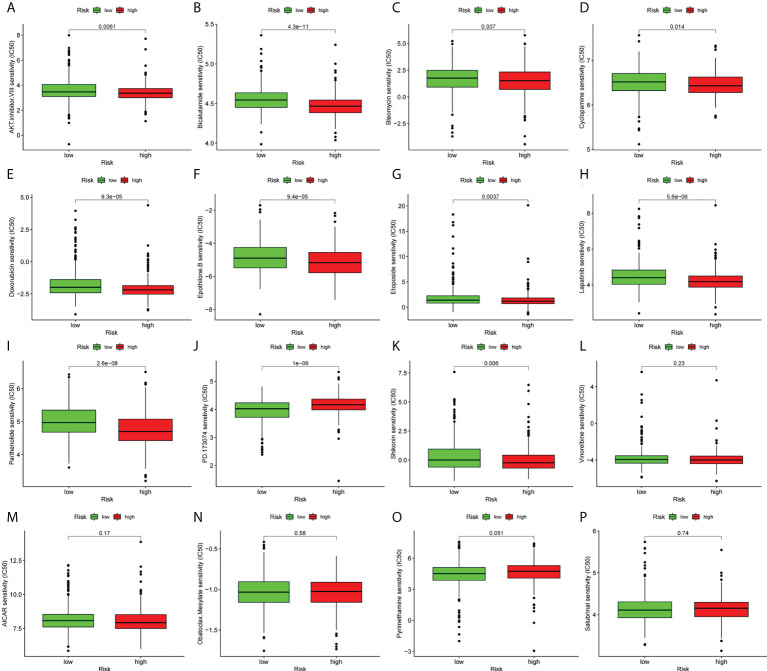
Prediction of response to common chemotherapeutic drugs between the low- and high-risk groups. **(A–P)** Patients in the high-risk group (n=267) exhibited higher estimated half-maximal inhibitory concentration compared with patients in the low-risk group (n=244).

Furthermore, the current study aimed to evaluate the potential of the lncRNA signature in advising on systemic therapies. The pRRophetic algorithm was used to evaluate the IC_50_ values of the drugs and to predict the effect of ferroptosis-related lncRNAs on chemotherapy response between the high- and low-risk groups. The analysis revealed that LUAD patients could be sensitive to 11 types of traditional anti-cancer drugs (**Figure 8**).

### Compare ROC and survival status with published signature in tumor microenvironment

**Figure 9:**
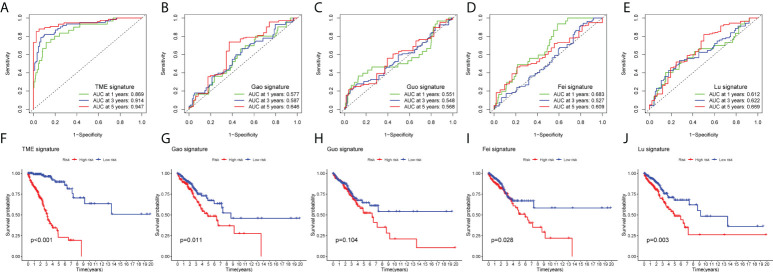
Compare the models among other’s signature. **(A–E)** AUC at 1, 3, 5 years for different models. **(F–J)** Significance of Survival State in Different Models.

To highlight the superiority of our model, we compared the published literature on ferroptosis-related lncRNAs, among which Gao et al. (32), Guo et al. (33), Fei et al. (34), Lu et al. (35). and our TME signature, the 5-year ROCs were 0.646, 0.568, 0.609, 0.669 and 0.947, respectively (**Figures 9A** **–** **E**). **Figures 9F** **–** **J** shown that the survival state of the above model. These results show that our signature has higher sensitivity and specificity than other models.

### Analysis of the expression of ferroptosis-related lncRNAs in LUAD

**Figure 10:**
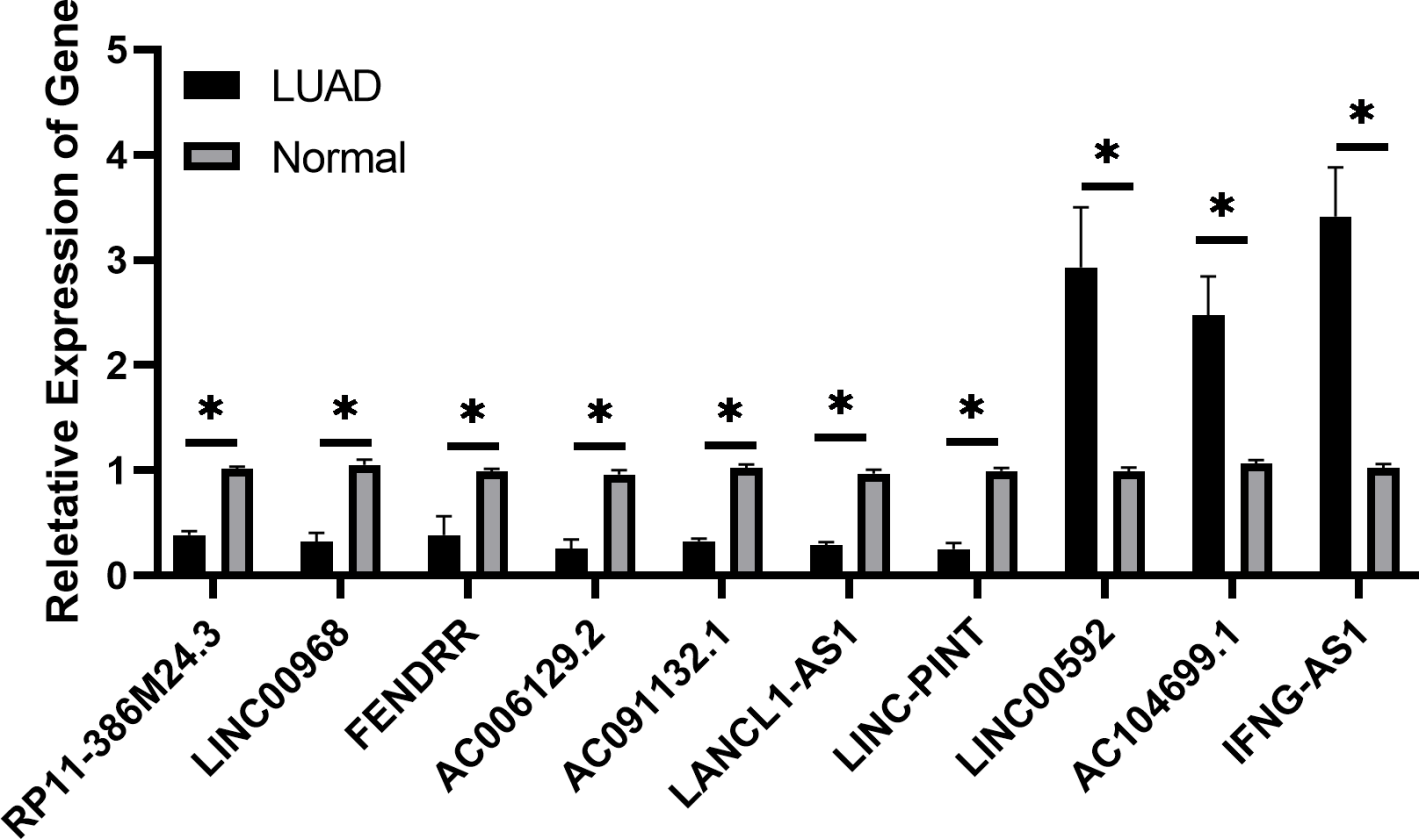
Expression of ferroptosis-related long non-coding RNAs in lung adenocarcinoma tissues by reverse transcription-quantitative PCR is shown. *P<0.05.

The expression levels of 10 candidate ferroptosis-related lncRNAs were determined by RT-qPCR in LUAD and paired normal samples. The results demonstrated that LINC00592, AC104699.1 and IFNG-AS1 were significantly upregulated in LUAD tissues, while RP11-386M24.3, FENDRR, AC091132.1, LANCL1-AS1, LINC-PINT, LINC00968 and AC006129.2 were notably downregulated (**Figure 10**). These above results were consistent with the results of the database analysis.

## Discussion

LUAD is the most common type of NSCLC. In recent years, despite the advances in screening, diagnosis and treatment, the pathogenesis of LUAD remains elusive due to its complex underlying genetic and molecular mechanisms (36). As emerging non-coding gene biomarkers, lncRNAs are gaining increasing attention and can play an essential role in the occurrence and progression of several types of cancer, including LUAD (37, 38). Ferroptosis is a recently identified type of programmed cell death, characterized by the excessive accumulation of iron-dependent reactive oxygen species and lipid peroxides, and is closely associated with pathophysiological processes in several diseases, including LUAD (39). However, ferroptosis-related lncRNAs capable of predicting the prognosis of patients with LUAD are still unknown. Therefore, the present study aimed to construct a prognostic model *via* identifying ferroptosis-related lncRNAs to predict LUAD and improve the OS of patients.

Correlation analysis was used to screen 2,420 ferroptosis-associated lncRNAs. Among them, 259 ferroptosis-related mRNAs were obtained. Univariate Cox regression analysis revealed 29 ferroptosis-associated lncRNAs with potential prognostic value in LUAD. Lasso regression analysis was used for dimensionality reduction to avoid overfitting, while multivariate Cox regression analysis was applied to construct a signature of 10 ferroptosis-related lncRNAs with the lowest Akaike Information Criterion (AIC) values. Furthermore, all patients were assigned to high- and low-risk groups based on the median risk score. Based on univariate and multivariate Cox regression analysis, risk score was identified as an independent risk factor for prognosis of patients with LUAD. The AUC values were then calculated to verify the distinguishing ability and accuracy of the lncRNA signature. Additionally, based on multivariate analysis a model was established, which could directly reflect the extent to which risk scores could exert an effect on predicting OS.

Previous studies demonstrated that LINC00592 could be a prognostic biomarker for disease free survival in gastric and cervical cancer (40, 41). In addition, another study revealed that IFNG-AS1 could enhance the secretion of IFN-γ in human natural killer cells (42). A previous study also showed that the down-regulated expression of lncRNA LINC00968 in LUAD was associated with the proliferation, migration and invasion (43). Emerging evidence has suggested that LANCL1-AS1 plays a key role in regulating NSCLC (44, 45). LANCL1-AS1 could be also considered as a novel target for treating patients with NSCLC (44). Al-Raawi et al. demonstrated that JARID2-AS1 could be involved in an auto-regulatory loop, m odulating the expression of JARID2, which in turn could be involved in differentiation processes *via* interacting and probably recruiting PRC2. However, the effects of CTD-2017D11.1, AC002117.1 and AC007036.4 lncRNAs remain unknown. Therefore, future studies focusing on the above lncRNAs are urgently needed to develop novel strategies for the diagnosis and treatment of LUAD.

Tumor-associated immune responses play an important role in cell infiltration and metastasis in the tumor microenvironment. In turn, lncRNAs and ferroptosis serve key regulatory roles in tumor-associated immune responses (46–48). Herein, immune-related GSEA revealed that immune system processes and metabolic pathways were markedly enriched in the low-risk group compared with the high-risk one, thus suggesting that low-risk patients could present ferroptosis-associated anti-tumor immune responses, eventually enhancing the survival rate of LUAD patients.

Recently, with the discovery of lncRNA functions, growing advancements have been achieved regarding the effects of ferroptosis on cancer therapy. However, the association between lncRNAs and ferroptosis remains to be fully investigated, especially in LUAD. In the present study, 10 lncRNAs associated with iron degeneration were identified using data obtained from TCGA. In addition, the implication of the above lncRNAs in immune responses and metabolic pathways were also explored.

However, the current study has some limitations. Firstly, only data obtained from TCGA was used to establish a ferroptosis-related lncRNA prognostic model and to evaluate its validity. In addition, the number of experiments on detecting the expression levels of the identified ferroptosis-associated lncRNAs in clinical samples and cell lines were limited. Therefore, further *in vitro* experiments are needed to fully elucidate the mechanisms underlying the effects of ferroptosis-related lncRNAs on LUAD.

## Conclusion

In the present study, signatures of 10 ferroptosis-associated lncRNAs with potential independent prognostic value that were associated with immune responses were identified in LUAD and validated. The results suggested that the above lncRNAs could serve as potential prognostic indicators and be considered as novel therapeutic strategies focusing on ferroptosis, thus improving the prognosis of patients with LUAD.

## Data availability statement

The original contributions presented in the study are included in the article/**Supplementary Material**. Further inquiries can be directed to the corresponding authors.

## Ethics statement

The studies involving human participants were reviewed and approved by the ethics committee of the Tangdu Hospital of Air Force Medical University. The patients/participants provided their written informed consent to participate in this study.

## Author contributions

XY, XL, and JH contributed to the conception and design of the study. YW, GL, XX, MX, ZW, ZM, YF, CS, and HD analyzed the data and wrote the manuscript. MP and PD collected and analyzed the data. All authors contributed to the article and approved the submitted version.

## Funding

The present study was supported by the National Natural Science Foundation of China (grant nos. 82173252, 81871866 and 81700007), the Shaanxi Social Development Science and Technology Key Project (grant no. 2016SF-308) and the Project of Tangdu Hospital, The Fourth Military Medical University (2018 Key Talents).

## Acknowledgments

We appreciate the free use of The Cancer Genome Atlas and FerrDb databases.

## Conflict of interest

The authors declare that the research was conducted in the absence of any commercial or financial relationships that could be construed as a potential conflict of interest.

## Publisher’s note

All claims expressed in this article are solely those of the authors and do not necessarily represent those of their affiliated organizations, or those of the publisher, the editors and the reviewers. Any product that may be evaluated in this article, or claim that may be made by its manufacturer, is not guaranteed or endorsed by the publisher.
