# Supplementary material for: Characterization and validation of a ferroptosis-related LncRNA signature as a novel prognostic model for lung adenocarcinoma in tumor microenvironment
Source: Front Immunol. 2022 Aug 9;13:903758. doi: 10.3389/fimmu.2022.903758 (PMC9395983; doi:10.3389/fimmu.2022.903758)
Supplement: Supplementary file 1 [file Table_1.docx]

Supplementary Table 1 ferroptosis-related mRNAs

| OFFICIAL GENE SYMBOL | Name |
| --- | --- |
| CYBB | cytochrome b-245 beta chain(CYBB) |
| MAFG | MAF bZIP transcription factor G(MAFG) |
| CDKN1A | cyclin dependent kinase inhibitor 1A(CDKN1A) |
| CDKN2A | cyclin dependent kinase inhibitor 2A(CDKN2A) |
| ATP5MC3 | ATP synthase membrane subunit c locus 3(ATP5MC3) |
| SRXN1 | sulfiredoxin 1(SRXN1) |
| SP1 | Sp1 transcription factor(SP1) |
| CDO1 | cysteine dioxygenase type 1(CDO1) |
| MIR9-3 | microRNA 9-3(MIR9-3) |
| KLHL24 | kelch like family member 24(KLHL24) |
| HIC1 | HIC ZBTB transcriptional repressor 1(HIC1) |
| SNX4 | sorting nexin 4(SNX4) |
| HIF1A | hypoxia inducible factor 1 subunit alpha(HIF1A) |
| SELENOS | selenoprotein S(SELENOS) |
| ATP6V1G2 | ATPase H+ transporting V1 subunit G2(ATP6V1G2) |
| SETD1B | SET domain containing 1B, histone lysine methyltransferase(SETD1B) |
| GLS2 | glutaminase 2(GLS2) |
| CEBPG | CCAAT enhancer binding protein gamma(CEBPG) |
| RIPK1 | receptor interacting serine/threonine kinase 1(RIPK1) |
| CISD1 | CDGSH iron sulfur domain 1(CISD1) |
| NOX5 | NADPH oxidase 5(NOX5) |
| ATG13 | autophagy related 13(ATG13) |
| ENPP2 | ectonucleotide pyrophosphatase/phosphodiesterase 2(ENPP2) |
| ACO1 | aconitase 1(ACO1) |
| LOC390705 | protein phosphatase 2 regulatory subunit B'', beta pseudogene(LOC390705) |
| SRC | SRC proto-oncogene, non-receptor tyrosine kinase(SRC) |
| BACH1 | BTB domain and CNC homolog 1(BACH1) |
| AGPAT3 | 1-acylglycerol-3-phosphate O-acyltransferase 3(AGPAT3) |
| LINC00472 | long intergenic non-protein coding RNA 472(LINC00472) |
| JDP2 | Jun dimerization protein 2(JDP2) |
| IREB2 | iron responsive element binding protein 2(IREB2) |
| HMGB1 | high mobility group box 1(HMGB1) |
| MAP1LC3A | microtubule associated protein 1 light chain 3 alpha(MAP1LC3A) |
| NCF2 | neutrophil cytosolic factor 2(NCF2) |
| GABARAPL2 | GABA type A receptor associated protein like 2(GABARAPL2) |
| PANX1 | pannexin 1(PANX1) |
| GCH1 | GTP cyclohydrolase 1(GCH1) |
| KEAP1 | kelch like ECH associated protein 1(KEAP1) |
| HMOX1 | heme oxygenase 1(HMOX1) |
| ACVR1B | activin A receptor type 1B(ACVR1B) |
| RRM2 | ribonucleotide reductase regulatory subunit M2(RRM2) |
| HNF4A | hepatocyte nuclear factor 4 alpha(HNF4A) |
| SLC7A11 | solute carrier family 7 member 11(SLC7A11) |
| PGD | phosphogluconate dehydrogenase(PGD) |
| AKR1C1 | aldo-keto reductase family 1 member C1(AKR1C1) |
| AKR1C2 | aldo-keto reductase family 1 member C2(AKR1C2) |
| PTGS2 | prostaglandin-endoperoxide synthase 2(PTGS2) |
| DDIT3 | DNA damage inducible transcript 3(DDIT3) |
| STAT3 | signal transducer and activator of transcription 3(STAT3) |
| MAP3K5 | mitogen-activated protein kinase kinase kinase 5(MAP3K5) |
| BAP1 | BRCA1 associated protein 1(BAP1) |
| PLIN2 | perilipin 2(PLIN2) |
| BID | BH3 interacting domain death agonist(BID) |
| TXNRD1 | thioredoxin reductase 1(TXNRD1) |
| FANCD2 | FA complementation group D2(FANCD2) |
| SLC38A1 | solute carrier family 38 member 1(SLC38A1) |
| LURAP1L | leucine rich adaptor protein 1 like(LURAP1L) |
| ACSL3 | acyl-CoA synthetase long chain family member 3(ACSL3) |
| AURKA | aurora kinase A(AURKA) |
| ACSL4 | acyl-CoA synthetase long chain family member 4(ACSL4) |
| LPIN1 | lipin 1(LPIN1) |
| PHKG2 | phosphorylase kinase catalytic subunit gamma 2(PHKG2) |
| JUN | Jun proto-oncogene, AP-1 transcription factor subunit(JUN) |
| DUOX1 | dual oxidase 1(DUOX1) |
| LONP1 | lon peptidase 1, mitochondrial(LONP1) |
| UBC | ubiquitin C(UBC) |
| BNIP3 | BCL2 interacting protein 3(BNIP3) |
| GABARAPL1 | GABA type A receptor associated protein like 1(GABARAPL1) |
| SAT1 | spermidine/spermine N1-acetyltransferase 1(SAT1) |
| NF2 | NF2, moesin-ezrin-radixin like (MERLIN) tumor suppressor(NF2) |
| GCLC | glutamate-cysteine ligase catalytic subunit(GCLC) |
| PIK3CA | phosphatidylinositol-4,5-bisphosphate 3-kinase catalytic subunit alpha(PIK3CA) |
| PROM2 | prominin 2(PROM2) |
| NFE2L2 | NFE2 like bZIP transcription factor 2(NFE2L2) |
| SQSTM1 | sequestosome 1(SQSTM1) |
| SCD | stearoyl-CoA desaturase(SCD) |
| NQO1 | NAD(P)H quinone dehydrogenase 1(NQO1) |
| HRAS | HRas proto-oncogene, GTPase(HRAS) |
| SCP2 | sterol carrier protein 2(SCP2) |
| FADS2 | fatty acid desaturase 2(FADS2) |
| SESN2 | sestrin 2(SESN2) |
| ALB | albumin(ALB) |
| OXSR1 | oxidative stress responsive kinase 1(OXSR1) |
| ULK1 | unc-51 like autophagy activating kinase 1(ULK1) |
| ARRDC3 | arrestin domain containing 3(ARRDC3) |
| ZFP69B | ZFP69 zinc finger protein B(ZFP69B) |
| FH | fumarate hydratase(FH) |
| HSF1 | heat shock transcription factor 1(HSF1) |
| GPT2 | glutamic--pyruvic transaminase 2(GPT2) |
| HILPDA | hypoxia inducible lipid droplet associated(HILPDA) |
| NNMT | nicotinamide N-methyltransferase(NNMT) |
| MIR4715 | microRNA 4715(MIR4715) |
| NOS2 | nitric oxide synthase 2(NOS2) |
| HSPA5 | heat shock protein family A (Hsp70) member 5(HSPA5) |
| ALOX12 | arachidonate 12-lipoxygenase, 12S type(ALOX12) |
| ALOX5 | arachidonate 5-lipoxygenase(ALOX5) |
| IL33 | interleukin 33(IL33) |
| ALOX12B | arachidonate 12-lipoxygenase, 12R type(ALOX12B) |
| HSPB1 | heat shock protein family B (small) member 1(HSPB1) |
| GOT1 | glutamic-oxaloacetic transaminase 1(GOT1) |
| ALOX15 | arachidonate 15-lipoxygenase(ALOX15) |
| ALOX15B | arachidonate 15-lipoxygenase type B(ALOX15B) |
| CHAC1 | ChaC glutathione specific gamma-glutamylcyclotransferase 1(CHAC1) |
| VDAC2 | voltage dependent anion channel 2(VDAC2) |
| PML | PML nuclear body scaffold(PML) |
| VEGFA | vascular endothelial growth factor A(VEGFA) |
| CA9 | carbonic anhydrase 9(CA9) |
| ATG5 | autophagy related 5(ATG5) |
| PLIN4 | perilipin 4(PLIN4) |
| KRAS | KRAS proto-oncogene, GTPase(KRAS) |
| BLOC1S5-TXNDC5 | BLOC1S5-TXNDC5 readthrough (NMD candidate)(BLOC1S5-TXNDC5) |
| DPP4 | dipeptidyl peptidase 4(DPP4) |
| ABCC1 | ATP binding cassette subfamily C member 1(ABCC1) |
| VLDLR | very low density lipoprotein receptor(VLDLR) |
| DDIT4 | DNA damage inducible transcript 4(DDIT4) |
| ATG16L1 | autophagy related 16 like 1(ATG16L1) |
| EGLN2 | egl-9 family hypoxia inducible factor 2(EGLN2) |
| PSAT1 | phosphoserine aminotransferase 1(PSAT1) |
| FLT3 | fms related receptor tyrosine kinase 3(FLT3) |
| WIPI1 | WD repeat domain, phosphoinositide interacting 1(WIPI1) |
| DRD4 | dopamine receptor D4(DRD4) |
| ZEB1 | zinc finger E-box binding homeobox 1(ZEB1) |
| DRD5 | dopamine receptor D5(DRD5) |
| CHMP6 | charged multivesicular body protein 6(CHMP6) |
| NRAS | NRAS proto-oncogene, GTPase(NRAS) |
| MIOX | myo-inositol oxygenase(MIOX) |
| SLC2A8 | solute carrier family 2 member 8(SLC2A8) |
| ATG7 | autophagy related 7(ATG7) |
| RB1 | RB transcriptional corepressor 1(RB1) |
| TSC22D3 | TSC22 domain family member 3(TSC22D3) |
| CISD2 | CDGSH iron sulfur domain 2(CISD2) |
| NGB | neuroglobin(NGB) |
| GDF15 | growth differentiation factor 15(GDF15) |
| DUSP1 | dual specificity phosphatase 1(DUSP1) |
| CHMP5 | charged multivesicular body protein 5(CHMP5) |
| CAPG | capping actin protein, gelsolin like(CAPG) |
| OTUB1 | OTU deubiquitinase, ubiquitin aldehyde binding 1(OTUB1) |
| GPX2 | glutathione peroxidase 2(GPX2) |
| GPX4 | glutathione peroxidase 4(GPX4) |
| SLC2A14 | solute carrier family 2 member 14(SLC2A14) |
| DNAJB6 | DnaJ heat shock protein family (Hsp40) member B6(DNAJB6) |
| CARS1 | cysteinyl-tRNA synthetase 1(CARS1) |
| XBP1 | X-box binding protein 1(XBP1) |
| DUOX2 | dual oxidase 2(DUOX2) |
| NOX4 | NADPH oxidase 4(NOX4) |
| NOX3 | NADPH oxidase 3(NOX3) |
| FTMT | ferritin mitochondrial(FTMT) |
| LAMP2 | lysosomal associated membrane protein 2(LAMP2) |
| RELA | RELA proto-oncogene, NF-kB subunit(RELA) |
| STMN1 | stathmin 1(STMN1) |
| IDH1 | isocitrate dehydrogenase (NADP(+)) 1(IDH1) |
| CAV1 | caveolin 1(CAV1) |
| ACSF2 | acyl-CoA synthetase family member 2(ACSF2) |
| NFS1 | NFS1 cysteine desulfurase(NFS1) |
| NCOA4 | nuclear receptor coactivator 4(NCOA4) |
| SNORA16A | small nucleolar RNA, H/ACA box 16A(SNORA16A) |
| LINC00336 | long intergenic non-protein coding RNA 336(LINC00336) |
| CXCL2 | C-X-C motif chemokine ligand 2(CXCL2) |
| TF | transferrin(TF) |
| YWHAE | tyrosine 3-monooxygenase/tryptophan 5-monooxygenase activation protein epsilon(YWHAE) |
| CBS | cystathionine beta-synthase(CBS) |
| SLC1A4 | solute carrier family 1 member 4(SLC1A4) |
| SLC40A1 | solute carrier family 40 member 1(SLC40A1) |
| SLC1A5 | solute carrier family 1 member 5(SLC1A5) |
| TFAP2C | transcription factor AP-2 gamma(TFAP2C) |
| RGS4 | regulator of G protein signaling 4(RGS4) |
| SLC2A1 | solute carrier family 2 member 1(SLC2A1) |
| ZFP36 | ZFP36 ring finger protein(ZFP36) |
| SIRT1 | sirtuin 1(SIRT1) |
| SLC2A3 | solute carrier family 2 member 3(SLC2A3) |
| PRDX6 | peroxiredoxin 6(PRDX6) |
| SLC3A2 | solute carrier family 3 member 2(SLC3A2) |
| TFR2 | transferrin receptor 2(TFR2) |
| TFRC | transferrin receptor(TFRC) |
| ZNF419 | zinc finger protein 419(ZNF419) |
| IFNG | interferon gamma(IFNG) |
| TXNIP | thioredoxin interacting protein(TXNIP) |
| TGFBR1 | transforming growth factor beta receptor 1(TGFBR1) |
| MT1G | metallothionein 1G(MT1G) |
| AIFM2 | apoptosis inducing factor mitochondria associated 2(AIFM2) |
| MIR137 | microRNA 137(MIR137) |
| ARNTL | aryl hydrocarbon receptor nuclear translocator like(ARNTL) |
| CS | citrate synthase(CS) |
| MT3 | metallothionein 3(MT3) |
| MAPK14 | mitogen-activated protein kinase 14(MAPK14) |
| NOX1 | NADPH oxidase 1(NOX1) |
| TRIB3 | tribbles pseudokinase 3(TRIB3) |
| EGFR | epidermal growth factor receptor(EGFR) |
| ATG3 | autophagy related 3(ATG3) |
| MTOR | mechanistic target of rapamycin kinase(MTOR) |
| PEBP1 | phosphatidylethanolamine binding protein 1(PEBP1) |
| EIF2S1 | eukaryotic translation initiation factor 2 subunit alpha(EIF2S1) |
| ANO6 | anoctamin 6(ANO6) |
| MIR17 | microRNA 17(MIR17) |
| SLC2A6 | solute carrier family 2 member 6(SLC2A6) |
| LPCAT3 | lysophosphatidylcholine acyltransferase 3(LPCAT3) |
| TP63 | tumor protein p63(TP63) |
| BRD4 | bromodomain containing 4(BRD4) |
| ISCU | iron-sulfur cluster assembly enzyme(ISCU) |
| ASNS | asparagine synthetase (glutamine-hydrolyzing)(ASNS) |
| PRKAA1 | protein kinase AMP-activated catalytic subunit alpha 1(PRKAA1) |
| TMBIM4 | transmembrane BAX inhibitor motif containing 4(TMBIM4) |
| TLR4 | toll like receptor 4(TLR4) |
| PRKAA2 | protein kinase AMP-activated catalytic subunit alpha 2(PRKAA2) |
| PRDX1 | peroxiredoxin 1(PRDX1) |
| FTH1 | ferritin heavy chain 1(FTH1) |
| CD44 | CD44 molecule (Indian blood group)(CD44) |
| AKR1C3 | aldo-keto reductase family 1 member C3(AKR1C3) |
| STEAP3 | STEAP3 metalloreductase(STEAP3) |
| ELAVL1 | ELAV like RNA binding protein 1(ELAVL1) |
| SOCS1 | suppressor of cytokine signaling 1(SOCS1) |
| SLC7A5 | solute carrier family 7 member 5(SLC7A5) |
| ALOXE3 | arachidonate lipoxygenase 3(ALOXE3) |
| FTL | ferritin light chain(FTL) |
| YY1AP1 | YY1 associated protein 1(YY1AP1) |
| ATF3 | activating transcription factor 3(ATF3) |
| MIR212 | microRNA 212(MIR212) |
| ATF4 | activating transcription factor 4(ATF4) |
| EIF2AK4 | eukaryotic translation initiation factor 2 alpha kinase 4(EIF2AK4) |
| TNFAIP3 | TNF alpha induced protein 3(TNFAIP3) |
| ATM | ATM serine/threonine kinase(ATM) |
| HAMP | hepcidin antimicrobial peptide(HAMP) |
| MAPK1 | mitogen-activated protein kinase 1(MAPK1) |
| MAPK3 | mitogen-activated protein kinase 3(MAPK3) |
| EMC2 | ER membrane protein complex subunit 2(EMC2) |
| HBA1 | hemoglobin subunit alpha 1(HBA1) |
| MAPK8 | mitogen-activated protein kinase 8(MAPK8) |
| MAPK9 | mitogen-activated protein kinase 9(MAPK9) |
| HSD17B11 | hydroxysteroid 17-beta dehydrogenase 11(HSD17B11) |
| MUC1 | mucin 1, cell surface associated(MUC1) |
| BECN1 | beclin 1(BECN1) |
| TUBE1 | tubulin epsilon 1(TUBE1) |
| SLC2A12 | solute carrier family 2 member 12(SLC2A12) |
| ATG4D | autophagy related 4D cysteine peptidase(ATG4D) |
| ANGPTL7 | angiopoietin like 7(ANGPTL7) |
| ULK2 | unc-51 like autophagy activating kinase 2(ULK2) |
| G6PD | glucose-6-phosphate dehydrogenase(G6PD) |
| MTDH | metadherin(MTDH) |
| HERPUD1 | homocysteine inducible ER protein with ubiquitin like domain 1(HERPUD1) |
| MIR30B | microRNA 30b(MIR30B) |
| IL6 | interleukin 6(IL6) |
| MIR6852 | microRNA 6852(MIR6852) |
| PCK2 | phosphoenolpyruvate carboxykinase 2, mitochondrial(PCK2) |
| EPAS1 | endothelial PAS domain protein 1(EPAS1) |
| WIPI2 | WD repeat domain, phosphoinositide interacting 2(WIPI2) |
| RPL8 | ribosomal protein L8(RPL8) |
| TP53 | tumor protein p53(TP53) |
| GABPB1 | GA binding protein transcription factor subunit beta 1(GABPB1) |
| MYB | MYB proto-oncogene, transcription factor(MYB) |
| FBXW7 | F-box and WD repeat domain containing 7(FBXW7) |
| HELLS | helicase, lymphoid specific(HELLS) |
